# The Role of Antimicrobial Peptides in Preventing Multidrug-Resistant Bacterial Infections and Biofilm Formation

**DOI:** 10.3390/ijms12095971

**Published:** 2011-09-16

**Authors:** Seong-Cheol Park, Yoonkyung Park, Kyung-Soo Hahm

**Affiliations:** 1 Research Center for Proteineous Materials, Chosun University, Gwangju 501-759, Korea; E-Mails: schpark9@hotmail.com (S.-C.P.); kshahm@chosun.ac.kr (K.-S.H.); 2 Department of Biotechnology, Chosun University, Gwangju 501-759, Korea; 3 Bioleaders Corporation, 559 Yongsan-Dong, Yusong-Ku, Daejeon, 305-500 Korea

**Keywords:** mode of action, lipopolysaccharide, quorum sensing, dental plaque, synergetic effect

## Abstract

Over the last decade, decreasing effectiveness of conventional antimicrobial-drugs has caused serious problems due to the rapid emergence of multidrug-resistant pathogens. Furthermore, biofilms, which are microbial communities that cause serious chronic infections and dental plaque, form environments that enhance antimicrobial resistance. As a result, there is a continuous search to overcome or control such problems, which has resulted in antimicrobial peptides being considered as an alternative to conventional drugs. Antimicrobial peptides are ancient host defense effector molecules in living organisms. These peptides have been identified in diverse organisms and synthetically developed by using peptidomimic techniques. This review was conducted to demonstrate the mode of action by which antimicrobial peptides combat multidrug-resistant bacteria and prevent biofilm formation and to introduce clinical uses of these compounds for chronic disease, medical devices, and oral health. In addition, combinations of antimicrobial peptides and conventional drugs were considered due to their synergetic effects and low cost for therapeutic treatment.

## 1. Introduction

Since penicillin was first discovered by Fleming in 1928, a large number of antibiotics have been identified, developed and clinically used in antimicrobial pharmatherapeutics. However, the widespread use of antibiotics was soon followed by the emergence of multidrug-resistant (MDR) microbes due to various reasons including abuse and the increasing use of antibiotics in the biomedical and agricultural fields. In addition to bacterial evolution, a number of patients in hospitals worldwide are currently suffering from superbugs such as vancomycin resistant enterococci (VRE), methicillin resistant *Staphylococcus aureus* (MRSA) and MDR bacteria. Indeed, from 1999 to 2005, the number of hospitalizations associated with MRSA infections increased by 119%, or ~14% per year [1]. In addition, the Center for Disease Control and Prevention (CDC) reported that 1.7 million people were nosocomially infected in hospitals in 2002 and 99,000 deaths were occurring annually in the United States due to drug-resistant microbes [2]. Moreover, as of early 2005 the number of deaths in the United Kingdom attributed to MRSA was estimated to be 3,000 per year [3].

Biofilms are sessile microbial communities of microbes that are adhered to various surfaces and encaged in a self-produced extracellular matrix, and have given rise to another problem in clinical therapeutics [4]. Specifically, bacterial cells growing in a biofilm are physiologically distinct from planktonic cells of the same bacteria and are embedded within a self-produced matrix of extracelluar polymeric substance (EPS) [4,5], which can increase antibiotic resistance by up to 1000 fold [6]. Infectious processes in biofilms are related to various routes such as urinary tract [7] and catheter infections [8] and the formation of dental plaque [9]. Among those, a total of 250,000 cases of catheter-associated blood stream infections that occur annually in the USA are attributed to a mortality rate of 12%~25% for each infection, with a treatment cost of $25,000 per episode [10].

Currently, many studies are being conducted to address the above problems, multidrug-resistant bacteria and biofilm formation. The results of these studies have led to antimicrobial peptides being considered as an alternative drug for conventional antibiotics. They have weak antimicrobial activity but potent and broad immune modulatory activity when the host organism is invaded by pathogenic microbes or viruses. Indeed many use the generic term “host defense” peptides [11–13]. They do not activate adaptive immunity, but rather increase the efficiency thereof through adjuvant activity. Since antimicrobial peptides were initially identified in frogs and insects in the 1980s (for example, cecropins [14], PGLa [15], magainins [16]), many additional peptides have been found and over 1200 have been isolated to date (http://aps.unmc.edu/AP/main.php and http://www.bbcm.units.it/~tossi/amsdb.html). Although the sequences and structures of these peptides are highly diverse, they have some common properties, including amphipathic secondary structures within membranes, a positive net charge under physiological conditions, small size, rapid binding to biological membranes, and usually the ability to kill invading microorganisms within minutes [17,18].

The mode of action of antibiotic peptides is not fully understood, but it is believed that their major targets are cytoplasmic membrane and intracellular molecules [19,20]. It is also believed that it is very difficult for bacteria to develop resistance to antimicrobial peptides because most kill bacterial cells quickly through their actions on the entire cytoplasmic membrane or can act through complex mechanism [12]. Although resistance for antimicrobial peptide has been reported, acquirement of resistance by changing the charge on surface molecules [21,22] or proteolytic cleavage by the release of extracelluar protease [23,24], is limited and also takes long periods when compared to conventional drugs. Although antimicrobial peptides are much more expensive than antibiotics, many studies have found that antimicrobial peptides act effectively in synergy with currently used antibiotics against multidrug-resistant bacteria [25–27] because they function through different mechanisms.

In this review, we will focus on the mode of action of antibiotics and antimicrobial peptides, their current use against multidrug-resistant bacteria, and recent findings regarding their use in the prevention of biofilms.

## 2. Use of AMPs in Preventing Multidrug-Resistant Bacteria

Major targets of antimicrobial peptides in bacterial cells can be divided into two cellular sites, the cell wall containing outer membrane and inner membrane and cytoplasm. Although the mechanisms inducing antibiotic-resistance are also diverse, the cellular action of antimicrobial peptides is separated from these mechanisms. For that reason, antimicrobial peptides have the potential for use in a unique antibiotic drug for combating or preventing the formation of multidrug-resistant bacteria.

### 2.1. Modes of Antibacterial Action

#### 2.1.1. Lipopolysaccharide (LPS) Neutralization or Disaggregation by Antimicrobial Peptides

LPSs are major components of the outer leaflet of the outer membrane in Gram-negative bacteria. LPSs consist of an O-specific chain that is highly variable in different bacterial strains, a core oligosaccharide, and lipid A [28]. LPSs are essential for bacterial growth and viability, but macrophages stimulated by LPS induce the release of pro-inflammatory cytokines (TNF-α, IL1 and IL6) into the blood, resulting in septic shock [29–31]. Accordingly, LPSs are an excellent target for antimicrobial peptides because they have the potential to both directly inhibit the growth of multidrug-resistant bacteria and to neutralize the action of released LPS due to its stimulation of immune cells.

Antimicrobial peptides generally bind to LPS through electrostatic interactions between their cationic amino acids (Lysine and Arginine) and head groups of LPS, and this complex is stabilized through hydrophobic interactions between the hydrophobic amino acids of the peptide and fatty acyl chains of LPS [32,33]. Since polymyxins, which are pentabasic decapeptide antibiotics, were discovered in *Bacillus polymyxa* [34], only two have been produced commercially, polymyxin B and E (colistin) [35]. Their action, which occurs via binding to lipid A of LPS and permeabilization of the outer membrane, is restricted to Gram-negative bacteria [36]. Sushi peptides, which are derived from Factor C (LPS-sensitive serine protease of the horseshoe crab coagulation cascade), disrupt LPS aggregates through detergent-like action and also have LPS-neutralizing activity [32,37]. Moreover, even though PMAP-23, which is a porcine myeloid antibacterial peptide composed of 23 residues that adopts a helix-hinge-helix structure in membrane-mimetic environments, showed a killing activity against a broad spectrum of microbial organisms, carboxyl terminus led to growth inhibition of *E. coli* via the interaction with outer membrane containing LPS [38]. Conversely, several AMPs prevent LPS-induced cytokine induction in macrophages, resulting in interruption of the development of septic shock in animal models [39–41].

Recently, the emergence of some bacteria with modifications of lipid A and LPS, such as lipid A acylation [42], aminoarabinose of lipid A [43], and myristylation of LPS [44] which are induced by PhoP/PhoQ and PmeAB regulatory systems, has resulted in antimicrobial peptides having reduced antibacterial activity. However, this antimicrobial peptide-mediated resistance occurs when bacteria surviving in the presence of antimicrobial peptide are conducted by repeated treatments during a very long-term. Moreover, the net negative surface charge decreased by these modifications reduces the electrostatic interaction with positively charged antimicrobial peptides. Within the limits of this interaction, it is expected that this process may be overcome if other antimicrobial peptides which do not interact with LPS or possess other modes of action in bacteria were substituted.

### 2.2. Cell Wall-Lipid II

Cell walls of Gram-positive bacteria are formed by peptidoglycan, which are composed of polymers of sugars and amino acids outside the plasma membrane [45]. Occasionally, inhibition of the production of peptidoglycan leads to resistance against antibiotics such as penicillin, which is inhibited via penicillin-binding proteins or transpeptidases [46,47]. MRSA is also related to the existence of the penicillin-binding protein 2a (PBP2a), which is not present in susceptible *S. aureus* [48,49]. Vancomycin resistance is caused by the production of depsipeptide d-Ala-d-Ala in the peptidoglycan [50]. Although a number of antimicrobial peptides have been shown to be active against MRSA and VRE, in this section, we focus on antibacterial peptides with unusual amino acids, which are known as lantibiotics, because many of them exert antibacterial action through the interaction with cell wall components.

Lantibiotics are ribosomally-synthesized and post-translationally modified peptides that contain an intramolecular ring structure. These compounds are produced by Gram-positive bacteria and exert potent inhibitory action against a wide-spectrum of bacteria [51]. These compounds are classified as either type-A or type-B, and damage the bacterial membrane and inhibit the production of enzymes, respectively [51]. Type-A lantibiotics include nisin [52], subtilin [53], epidermin [54], and Pep5 [55], while type-B include mersacidin [56] and cinnamycin [57]. The most well-known lantibiotic is nisin, which was isolated from *Lactococcus latis* [52] and is used as a food preservative worldwide [58]. It was initially shown that nisin forms complexes with lipid I and lipid II, and then inhibits cell wall biosynthesis [59–61]. Recently, it was shown that nisin can produce short-lived pores that cause the cytoplasmic membrane to be permeable [51,60]. Subtilin permeabilizes lipid membranes in a lipid II-dependent manner and binds bactoprenyl pyrophosphate [62]. Type-B lantibiotics have been shown to inhibit the biosynthesis of cell walls by complexing lipid II, which is essential for the growth and replication of bacteria [63]. Mersacidin does not influence the C-terminal d-Ala-d-Ala moiety of the lipid intermediate, which induces vancomycin resistance [64].

### 2.3. Alteration of Membrane Potential or Induction of Membrane Permeabilization

Two major mechanisms of multidrug-resistance are phenotypic alteration of microbes under specific growth conditions, such as biofilms, and reduction of drug accumulation into microbes through limited uptake or pumping out drugs by multidrug-resistant proteins (MDRPs) [65–68]. Mode of action of antimicrobial peptides in the cytoplasmic membrane is considered to be more important than other targets. Furthermore, antimicrobial peptides must permeate the cell wall and cytoplasmic membrane to reach their intracellular targets, which are nucleic acids and functional proteins [69].

**Figure 1:**
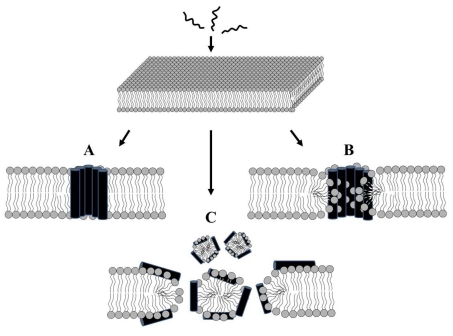
Three typical modes of action of antimicrobial peptides against cytoplasmic membranes. (**A**) barrel-stave model; (**B**) toroidal pore model; (**C**) carpet model.

Although the exact mechanisms of antimicrobial peptides are not fully understood, they are known to cause the efflux of intracellular materials by disrupting the cytoplasmic membrane via either pore formation through a barrel-stave [70] or a toroidal pore [71,72] mechanism, or through a nonpore carpet-like mechanism [73] (Figure 1). In the barrel-stave model (Figure 1A), a variable number of channel-forming peptides are positioned in a barrel-like ring around an aqueous pore. Generally, the peptide, which is most likely in monomeric form, must associate with the surface of the membrane prior to insertion, and the hydrophobic region of the bound peptides is then inserted into the membrane to a depth that varies depending on the hydrophobicity of the membrane outer leaflet. When the bound peptide reaches a threshold concentration, peptide monomers self-aggregate and are inserted deeper into the hydrophobic membrane core. The hydrophobic faces of the peptides then align and face the hydrophobic lipid core region, whereas their hydrophilic faces form the interior region of a water-filled pore [71,74]. This type of transmembrane pore is induced by alamethicin [75] and ceratotoxin [76].

In the carpet model (Figure 1C), antimicrobial peptides accumulate on the membrane surface, where they are electrostatically bound to the anionic phospholipid head groups, carpeting the membrane surface at numerous sites. When a threshold peptide concentration is reached, membrane disruption occurs in a detergent-like manner that does not involve pore formation, and the peptides do not necessarily insert into the hydrophobic core [71]. This model explains how cecropin P1 [77] and caerin 1.1 [78] disrupt membranes.

A toroidal pore model (Figure 1B) has been suggested for magainin [79,80], cathelicidin [81,82], and HPA3 [83,84]. In this model, antimicrobial peptides bound to the phospholipid headgroup regions of the bilayer induce a high-curvature fold in the bilayer, enabling the two leaflets of the bilayer to communicate directly at a torus lined by the leaflets [85]. This differs from the barrel-stave model in that antimicrobial peptides are associated onto the lipid head groups even when they are perpendicularly inserted into the lipid bilayer [74]. Recently, Han *et al.* [86] directly observed magainin action on artificial vesicles using cryo-transmission electron microscopy (TEM) and an image analysis technique. They proposed that magainin-induced pores in lipid vesicles possess a mean diameter of approximately 8 nm. A pore formed by HPA3 peptide was also observed under TEM in our study [83]. Other groups demonstrated that melittin formed a pore via the toroidal mechanism, even though it was dependent on the lipid material properties and peptide concentrations [87,88].

### 2.4. Inhibition of Cytoplasmic Proteins Related to Cell Division or Survival

Although most antimicrobial peptides primarily contribute to membrane perturbation, some antimicrobial peptides can penetrate the bacterial cytosol through a flip-flop mechanism or outer membrane protein forming channel. Among these, proline-rich antibacterial peptides such as pyrrhocoricin [89], apidaecin [90] and drosocin [91] have been shown to kill bacterial species by binding to the multi-helical lid region of the bacterial DnaK heat shock protein, which plays an essential role in the initiation of chromosomal DNA replication in an ATP-dependent manner with the other protein, DnaJ. The C-terminus of pyrrhocoricin was allowed to penetrate into cytosol of bacteria and the N-terminus responded to inhibit the ATPase activity of DnaK protein [89]. Microcin B17, which is ribosomally synthesized antimicrobial peptides from *Enterbacteriaceae*, is also believed to inhibit DNA replication by targeting DNA gyrase [92].

### 2.5. Inhibition of Macromolecular Synthesis through Interaction with Nucleic Acids

It has been suggested that inhibition of intracellular processes by certain antimicrobial peptides that penetrate bacterial cells, such as buforin II [93], PR-39 [94], indolicidin [95], and tPMP [96], may contribute to inhibition of the growth of bacterial cells or lead to cell death. Cho *et al.* [97] found that buforin II, a 21-amino acid peptide derived from the Asian toad, *Bufo bufo gargarizans*, kills bacteria through interaction with nucleic acids without membrane permeabilization, although further investigation is needed to identify other interactions with as yet unidentified intracellular targets. PR-39, which was isolated from the small intestine of the pig, required a lag period of about 8 min to penetrate the outer membrane, after which it rapidly killed growing *E. coli* cells via a mechanism that stops protein and DNA synthesis [94]. In the case of indolicidin, although it induced permeabilization of the bacterial membrane, it did not lyse the bacterial cells. Its lethal concentration allowed their filamentous morphology by inhibition of DNA synthesis in *E. coli* cells [95], and it was also found to bind specifically to DNA rather than RNA [98].

## 3. Synergetic Effects between Antimicrobial Peptides and Clinically used Antibiotics

The combined administration of antibiotics has gained interest because it often results in a synergistic antibacterial effect, which enables the dose of the individual drugs to be reduced [99]. In addition, certain combination therapies have prevented the development of drug-resistance in bacteria [100,101]. A membranolytic action of antimicrobial peptide is expected to produce synergetic effects when administered in combination with conventional antibiotics, and several studies have reported such findings [27,102,103]. Cirioni *et al*. [103] compared the synergies of magainin II and cecropin A administered with or without rifampicin against MDR *Pseudomonas aeruginosa* strains both *in vivo* and *in vitro* and found significant reductions in bacterial multiplication, LPS and TNF-α secretion in plasma and mortality. This finding suggested that the membrane-permeabilizing activity of peptides allows rifampicin to gain access to its intracellular target. On the other hand, synergistic effects between tachyplesin III peptide and imipenem are more effective *in vivo* mouse model of sepsis than *in vitro* study [104]. Our research also showed that P5 peptide is synergistic in combination with isepamicin against antibiotic-resistant *P. aeruginosa* from patients with cholelithiasis, but not with cefpiramide [105]. It suggested that P5 assisted penetration of isepamicin, which is an inhibitor of protein synthesis, into isepamicin-resistant strains because P5 exerted membranolytic action against bacteria. However, cefpiramide, which inhibits bacterial cell wall biosynthesis, was not synergetic. This proposed that membrane-acting peptides were effective not β-lactam but aminoglycoside antibiotics in combination due to modes of their action.

## 4. *In Vivo* Application of Antimicrobial Peptides

To data, large numbers of antimicrobial peptides have been identified in nature and designated *de novo*, and many of these have been confirmed to have potent antibacterial activity *in vitro*. However, most of clinical trials have attempted to topical applications, not to systemic applications (parenteral and oral). There are several obstacles to the use peptide therapeutic at required sites in the body through topical or oral dosing routes. These include the degradation of peptides by intestines, tissues, and serum protease and reduced half-life of small peptides through clearance by the kidneys [106].

Prior to the increment of circulation half-life, amino acids of antimicrobial peptide must be altered to be resistant against proteases or peptidases in serum or tissues. Many naturally isolated peptides have cationic amino acids, lysine and argine, which are easily cleaved by trypsin [107,108]. Moreover, chymotrypsin and elastase, which are proteases synthesized by pancreatic acinar cells and secreted in the small intestine [109], are responsible for cleaving peptide bonds in hydrophobic (phenylalanine, tryptophan, and tyrosine) and small amino acids (alanine, glycine, and valine), respectively [110]. To overcome the proteolytic cleavage of peptides, several trials have been conducted to evaluate the following: substitution of L-amino acids by d-amino acids [111,112], cyclization [113,114], conjugation of fatty acids [115], substitution by peptoids [116,117], use of fluorinated amino acids [118], beta-peptide [119], and acylation [120]. As novel candidates, although lantibiotics were mostly employed in food preservation, type-B is another prospective candidate in biomedical application against infections of MDR bacteria due to its resistance to proteolytic degradation [51]. Specifically, mersacidin has been shown to eradicate MRSA colonization in a mouse rhinitis model [121], and its *in vivo* efficacy is better against *Streptococcus pyogenes* than that of vancomycin [122].

Another problem involved in the preclinical development of antimicrobial peptides is that they are rapidly adsorbed in the kidneys during circulation due to their small size [105]. Several strategies to extend the length of the peptides for retardation of excretion through the kidney have been proposed. One typical method is attachment of polyethylene glycol (PEG), which is widely used to prolong serum half-life [123–125]. However, as with other bioactive peptides, longer PEGylation of antimicrobial peptides is unfavorable for *in vitro* activity, even though it enhances the circulating lifetime and decreases cytotoxicity [126,127]. Despite this, shorter PEGylation was found to enable retention of the *in vitro* antimicrobial activity of the model peptide and improved activity in the presence of serum in an *ex vivo* assay when compared to unPEGylated peptide [128]. Additionally, proteolytic degradation was reduced using this method. Nevertheless, length limitation of PEG and discovery of other methods requires further study to enable enhancement of both half-life time and antimicrobial activity *in vivo* prior to clinical trials.

## 5. Clinical Development of Antimicrobial Peptides

Several antimicrobial peptides are being evaluated in preclinical and clinical trials with limited applications. For example, Omeganan/MX-226, which is an indolicidin analogue, has recently completed phase III trials the prevention of catheter-related local and bloodstream infection, but was dropped for development [129–131]. Additionally, pexiganan/MSI-78 has completed phase III clinical trials in the prevention of diabetic foot ulcers [131,132] and plectasin is a fungal defensin peptide that exerts bactericidal action against drug-resistant bacteria and is currently in the preclinical phase [132,133]. Opebacan, which is a human bactericidal/permeability-increasing protein derivative, has reached the phase II clinical trial for endotoxemia in hematopoetic stem cell transplant recipients [131,134]. Iseganan/IB-367 from pig protegrin-1 has failed in the prevention of oral mucositis because it did not have a comparative advantage to existing therapeutics [132]. Although several antimicrobial peptides are progressing to commercial development, records of clinical trials for antimicrobial peptides have been restricted to topical applications [132].

## 6. Use of AMPs in Preventing Biofilm

### 6.1. Biofilm Formation

Extended cultivation of bacterial cells results in adherence to animal tissues and inorganic materials [135]. This, in turn, allows the formation of a biofilm, which is a multilayered community of sessile bacterial cells. Biofilms provide a survival advantage over planktonic or free-floating bacteria by enhancing nutrient trapping and colonization [136]. Currently, biofilms are a widespread problem in hospitals and healthcare facilities. Indeed, the United States National Institutes of Health found that 80% of chronic infections are related to biofilms [4]. Moreover, many studies have found that biofilms are associated with dental plaque [137,138], endocarditis [139], lung infection [140,141], and infection through medical devices [142].

**Figure 2:**
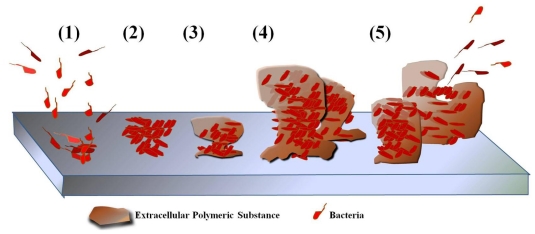
General overview of bacterial biofilm development. (**1**) reversible adsorption of bacteria; (**2**) irreversible attachment of bacteria; (**3**) production of extracelluar polymeric substance and biofilm growth; (**4**) maturation; (**5**) dispersion. After dispersion of the biofilm, bacteria move to other organs, tissues, or surfaces and a new biofilm is formed via stages (**1**)**–**(**5**).

Biofilm-formation by bacteria is achieved via responses to various factors, such as nutritional cues, cellular recognition of attachment sites on the surface, exposure to sublethal concentrations of antibiotics, and environment stresses [143,144]. As shown in Figure 2, biofilm-formation is generally initiated by the attachment of planktonic cells to a surface through weak van der Waals forces (Figure 2(1)), and the colonists are anchored tightly or irreversibly by pili (Figure 2(2)). To facilitate the arrival and attachment of other planktonic cells, the initial cells construct various adhesion sites and the matrix (Figure 2(3)). Bacterial cells are then embedded within this matrix of extracelluar polymeric substance (EPS), which is composed of extracelluar DNA, proteins, lipids, and polysaccharides with various configurations [145]. These components are very important targets for overcoming both biofilms and drug-resistant bacteria [146]. During colonization, some bacteria can communicate through a quorum sensing (QS) system [147,148] via small molecules called autoinducers and controls collective behaviors, such as bioluminescence, virulence factor production, and biofilm formation [149–151]. Autoinducers in Gram-negative and -positive bacteria were known to acyl-homoserine lactone molecules and oligopeptides, respectively. It is currently considered a good target for preventing biofilm infection. Subsequently, the grown or developed biofilm provides increased antibiotic-resistance to bacterial colonies through cell division and recruitment (Figure 2(4)). Later, the developed biofilms are dispersed and the bacteria move to other surfaces, such as organs, tissues, and medical devices (Figure 2(5)), where the biofilm formation process occurs again.

### 6.2. Applications to Prevent or Remove Biofilms

Two main concepts in the prevention of biofilms are dispersion of the biofilm EPS and eradication of the bacteria embedded in the EPS. Typically, lethal or inhibiting concentrations of antibiotics are significantly increased by up to 1000-fold against biofilm bacteria because they are unable to translocate into EPS and therefore do not reach the bacterial cells. In contrast, antimicrobial peptides are believed to have the potential for use as anti-biofilm agents due to their different mechanisms, which include membrane-disrupting action, functional inhibition of proteins, binding with DNA, and detoxification of polysaccharides (lipopolysaccharide and lipoteichoic acid). The EPSs of biofilms contain considerable amounts of polysaccharides, proteins, nucleic acids and lipids [152]. For example, certain antimicrobial peptides can be transferred in biofilm EPS through holes or pores formed in the lipid component of the EPS, while others can disperse biofilms.

#### 6.2.1. *In Vitro* Anti-Biofilm Activity of Antimicrobial Peptides against Biofilm of MDR Bacteria

*Pseudomonas aeruginosa* is the significant pulmonary pathogen affecting patients with cystic fibrosis [153], and this organism forms a biofilm on medical devices and tissues. LL-37, a human cationic host defense peptide, showed a potent inhibitory activity in biofilm formation at a concentration of 0.5 μg/mL against *P. aeruginosa* biofilm and reduced pre-grown biofilms [154]. It was also demonstrated that these effects were achieved by decreasing the attachment of bacterial cells onto the surface, stimulating twitching motility mediated by type IV pili, and down-regulating the genes related to the QS system [154]. LL-37 also inhibited both the attachment action and development of biofilms by Staphylococcus epidermidis, being commensal in human skin and mucous membrane [155]. Moreover, LL-37 potently inhibited the growth of planktonic cells and biofilm formation against *Francisella novicida*, which causes the disease tularemia [156]. Dashper *et al*. reported that kappacin, nonglycosylated κ-casein (109-169), showed a significant reduction of *Streptococcus mutans* biofilm in the presence of ZnCl_2_. In addition, systematic replacement of an N-terminal amino acid with fatty acids [157] or conjugation of fatty acids in N-terminus of synthetic short peptide [158] leads to enhanced antibiofilm activity.

#### 6.2.2. Anti-Biofilm Activity in Medical Devices

Recently, the beneficial effects on the survival and quality of life of patients have led to increased use of medical implants [159]. However, medical device-related infections are often serious because contaminating bacteria on the surface of these devices can form biofilms with dense layers that are very difficult to completely remove. Currently available antibiotics fail to eradicate such infections because they are inactive in the presence of biofilms or MDR bacteria [160]. Therefore, many researchers are suggesting that antimicrobial peptide administered alone or in combination with other molecules may be able to solve this problem.

Yoshinari *et al*. investigated that the adsorption of conjugated lactoferricin onto titanium surface was enhanced in the presence of hexapeptidic titanium-binding peptide and the attachment of *Porphyromonas gingivalis* was decreased onto this peptide-modified specimen, indicating that surface-modification with peptides can be presented as preventing method for biofilm formation on medical devices [161]. Melimine, which is a non-hemolytic hybrid peptide between melittin and protamine, did not induce resistance against *S. aureus* or *P. aeruginosa* during repeated passage in sub-minimal inhibitory concentrations, and reduced bacterial adhesion to contact lenses to which it was covalently linked [162]. Furthermore, silicone hydrogel lenses with melamine reduced contact lens-induced acute red eye in the *P. aeruginosa* guinea pig model and prevented contact lens induced peripheral ulcers in a *S. aureus* rabbit model [163]. Citropin 1.1, isolated from the green tree frog *Litoria citropa*, has potent anti-biofilm activity and showed enhanced activity against *S. aureus* biofilm when administered in combination with rifampin and minocycline [164]. The treatment of central venous catheters pre-treatedwith citropin 1.1 peptides and/or antibiotics significantly reduced bacterial counts of biofilm in a *S. aureus* infection rat model [160].

#### 6.2.3. Anti-Biofilm Activity against Oral Plaque

Dental plaque is a complex biofilm community that forms on the teeth and oral tissues of shedding and retentive surfaces [165,166]. Dental plaque develops under a variety of conditions and environments, and is composed of different bacterial species [167,168]. Oral biofilms cause dental cavities and periodontal diseases, such as gingivitis and chronic periodontitis [169,170]. Various therapeutic approaches have been investigated to prevent or remove oral biofilm, here we introduce the applications of antimicrobial peptides.

Gingival epithelial cells express antimicrobial peptides such as human beta-defensin-2 (hBD-2), psoriasin (S100A7), and ribonuclease 7 (RNase 7), which play important roles in innate immunity, through biofilm stimulation [171]. Expression of these peptides can be genetically regulated on epithelial cells. Another study showed that combined treatment with chlorhexidine and bacteriocin PsVP-10 synergistically reduced the number of the biofilm-forming bacteria, *Streptococcus mutans* [172]. Lactoferrin (LF) which exists in saliva and gingival crevicular fluids is related to host defense against oral pathogens [173]. The initial attachments of *Streptococcus gordonii* and *S. mutans* forming biofilm in oral cavity were inhibited in the presence of LF [174]. It was recently investigated that LF was able to inhibit planktonic growth of *Porphyromonas gingivalis* and *Prevotella intermedia*, which make biofilm in the subgingival plaque, and to suppress biofilm formation at a low concentration (≥8 μg/mL) [173]. LF alone or in combination with antibiotics also showed a reduction of pre-forming biofilm [173]. Moreover, in small-scale clinical trial, patients administered a tablet with 0.3 g of bovine LF for 3 months had an effect on reduction of bacterial numbers in the subgingival plaque [175]. Leung *et al*. proposed an interesting approach in which a chewing gum containing both KSL-W synthetic peptide and cetylpyridinium chloride displayed a dose-dependent reduction against a biofilm of human salivary bacteria [170]. Gum formulation with this combination was proposed to be used as an antiplaque agent or adjunct for oral hygiene.

#### 6.2.4. Others

Another approach to inhibiting biofilm formation is the use enzymes that can degrade the EPS of biofilm and detach established biofilm colonies. Moreover, biofilm-dispersing enzymes administered in combination treatment with antimicrobial agents will allow them to kill bacteria embedded in EPS [176]. Kaplan *et al*. suggested that deoxyribonuclease I and glycoside hydrolase dispersin B are useful as anti-biofilm agents due to the dispersing action of EPS layers on medical devices [177,178]. In addition, therapeutic treatment of combination treatments with antimicrobial peptides may result in significant synergetic-effects against MDR bacteria and the formation of biofilms.

## 8. Conclusions

Antimicrobial peptides can be the next generation of antibiotics for combating multi-drug resistant and/or biofilm forming bacterial infections. These peptides have a strong potential for application as nanofilms or other coating materials for surgical devices, including catheters. Even though there are drawbacks to the use of peptides as therapeutics, such as low bioavailability and high cost, these obstacles may be overcome since a great deal of effort is being conducted to circumvent the problems associated with various methods including the use of d- or unnatural amino acid, formulation, recombinant DNA expression of peptides, addition of fatty acyl chains to short peptides. Therefore, it is expected that antimicrobial peptides will become the drugs of choice for emerging bacterial infections in the future.
